# THE 6-MINUTE WALK TEST AND OTHER ENDPOINTS IN DUCHENNE MUSCULAR DYSTROPHY: LONGITUDINAL NATURAL HISTORY OBSERVATIONS OVER 48 WEEKS FROM A MULTICENTER STUDY

**DOI:** 10.1002/mus.23902

**Published:** 2013-06-26

**Authors:** Craig M Mcdonald, Erik K Henricson, R Ted Abresch, Julaine M Florence, Michelle Eagle, Eduard Gappmaier, Allan M Glanzman, for the PTC124-GD-007-DMD STUDY GROUP, Robert Spiegel, Jay Barth, Gary Elfring, Allen Reha, Stuart Peltz

**Affiliations:** 1 Department of Physical Medicine and Rehabilitation, University of California Davis School of MedicineDavis, California, 95817, USA; 2 Department of Neurology, Washington University School of MedicineSt. Louis, Missouri, USA; 3 Institute of Genetic Medicine, Newcastle UniversityNewcastle upon Tyne, UK; 4 Department of Physical Therapy, University of Utah School of MedicineSalt Lake City, Utah, USA; 5 Department of Physical Therapy, Children's Hospital of PhiladelphiaPhiladelphia, Pennsylvania, USA; 6 PTC Therapeutics, South PlainfieldNJ, USA

**Keywords:** ambulation, Duchenne muscular dystrophy, dystrophinopathy, myometry, natural history, prediction of loss of function, timed function tests, 6-minute walk test

## Abstract

*Introduction*: Duchenne muscular dystrophy (DMD) subjects ≥5 years with nonsense mutations were followed for 48 weeks in a multicenter, randomized, double-blind, placebo-controlled trial of ataluren. Placebo arm data (*N* = 57) provided insight into the natural history of the 6-minute walk test (6MWT) and other endpoints. *Methods*: Evaluations performed every 6 weeks included the 6-minute walk distance (6MWD), timed function tests (TFTs), and quantitative strength using hand-held myometry. Results: Baseline age (≥7 years), 6MWD, and selected TFT performance are strong predictors of decline in ambulation (Δ6MWD) and time to 10% worsening in 6MWD. A baseline 6MWD of <350 meters was associated with greater functional decline, and loss of ambulation was only seen in those with baseline 6MWD <325 meters. Only 1 of 42 (2.3%) subjects able to stand from supine lost ambulation. *Conclusion*: Findings confirm the clinical meaningfulness of the 6MWD as the most accepted primary clinical endpoint in ambulatory DMD trials.

Duchenne muscular dystrophy (DMD) is a disabling and life-threatening X-linked genetic disorder caused by defects in the gene for dystrophin that results in a progressive loss of functional muscle fibers and weakness with stereotypic functional consequences affecting mobility, progressive musculoskeletal deformities, upper limb impairment, impaired airway clearance and ventilation, cardiomyopathy, and premature death.^1^,^2^

The past several years have seen increased interest by biopharmaceutical companies in conducting ground-breaking research and development into novel treatment agents for DMD. There is considerable hope that new disease-modifying therapies will slow disease progression, stabilize or improve function, and maintain quality of life. The DMD population targeted by such trials features patients who are old enough to be assessed reliably and still have sufficient muscle fibers available for therapeutics to demonstrate a measurable benefit over the typical 1-year clinical trial duration.

Bipedal ambulation constitutes an almost uniquely human characteristic and is the most commonly performed physical activity by humans.^3^ Previously, we modified the American Thoracic Society version of the 6-minute walk test (6MWT) for DMD and validated the 6-minute walk distance (6MWD) as a measure of disease progression in ambulatory DMD patients.^4^–^6^ Given that ambulatory compromise is such an important component of the DMD disease process, improvement in the 6MWD with treatment relative to placebo constitutes clear evidence of therapeutic value. Thus, rather than being a surrogate endpoint, a change in the 6MWD can be defined as an important clinically meaningful endpoint that directly assesses how an ambulatory DMD patient functions.^7^ Evidence documenting the reliability of the 6MWT and the concurrent validity, sensitivity, and clinical meaningfulness of longitudinal changes in the 6MWD^4^–^6^,^8^,^9^ has led to its common use as the primary clinical endpoint in ambulatory DMD trials worldwide.

Because loss of muscle function in DMD occurs against the background of normal childhood growth and development associated with increases in stride length, children with DMD who are <7 years of age may show increases in 6MWD over ∽1 year despite progressive muscular impairment.^6^,^9^,^10^ Older DMD subjects demonstrate variable rates of decline in 6MWD. These findings emphasize the importance of age as a stratification factor in DMD clinical trials using the 6MWD. In addition, Henricson and colleagues^10^ showed that normalizing the data by converting 6MWD to a percent predicted 6MWD value may help to distinguish normal growth and development from disease-related progression and treatment effects. The results accumulated over the last several years have shown that the 6MWT offers advantages in DMD as an integrated global measure of ambulatory function that is influenced by lower extremity strength, biomechanical inefficiencies, endurance, and cardiorespiratory status.^11^

Longitudinal 6MWD natural history data provide information on how DMD patient ambulation changes over time (change scores), as well as an understanding of how the variability of the 6MWT (standard deviation) changes over time. This information can be used in clinical trial design for novel therapeutics. Such data for the change score and the standard deviation (SD) of the change score allow for more accurate statistical powering of clinical trials. Furthermore, as we gain understanding of the natural history of DMD we will ultimately be able to use these and future data for patients who receive standard-of-care treatment as historical controls. This will allow comparisons of treated subjects with historical controls for determination of efficacy in trials targeting rare populations with genetic-based treatments.

To determine the clinical relevance of a treatment effect for a given endpoint one must know what constitutes a minimal clinically important difference (MCID). For the 6MWD applied to DMD this has been estimated to be ∽30 m using 2 statistical distribution approaches.^11^ Knowledge of the MCID difference in 6MWD and the relevant variability of the change score make it possible to design clinical trials of appropriate power and size.

The aim of this report of placebo-treated patients from the phase 2b ataluren clinical trial (PTC Therapeutics)^12^,^13^ is to: (1) determine the contemporary natural history of common clinical measures of disease progression in ambulatory DMD subjects [6MWD, timed function tests (TFTs), and quantitative strength using hand-held myometry]; (2) estimate the probability of clinically meaningful disease progression (defined as persistent 10% worsening in 6MWD) and evaluate baseline predictors of subsequent 10% worsening; and (3) assess the relationship between baseline 6MWD and future loss of ambulation.

## METHODS

### Overall Study Design and Procedures

The natural history data were derived from the placebo arm of a phase 2b, international, multicenter, randomized, double-blind, placebo-controlled, dose-ranging study to evaluate the efficacy and safety of ataluren in ambulatory male patients aged ≥5 years with nonsense mutation DMD. The study comprised a 6-week screening period and a 48-week blinded study drug treatment period. Patients were stratified by age (<9 or ≥9 years), use of glucocorticoids (yes or no), and baseline 6MWD (≥350 or <350 m), and were randomized 1:1:1 to receive ataluren, at either a high or low dose, or matching placebo, daily for 48 weeks. Evaluations were performed at screening, baseline, and every 6 weeks for 48 weeks.

### Participants

The study included DMD patients as described previoiusly.^11^ Institutional review boards/institutional ethics committees and health authorities approved the study protocol. All parents/participants provided signed informed consent/assent before study initiation.

### Outcome Measures

The following outcome measures were obtained during screening, at baseline, and every 6 weeks during treatment.

#### 6-Minute Walk Test/6-Minute Walk Distance

Ambulation was assessed via the 6MWT following standardized procedures developed at University of California, Davis,^7^ by measuring the 6MWD in meters. The specific details of the modifications to the 6MWT, reliability, and validity in DMD were reported previously.^5^,^6^,^11^

#### Timed Function Tests

TFTs included time taken to stand from a supine position, time taken to run/walk 10 m, and time taken to climb or descend 4 standard-size stairs.^14^–^22^ TFTs provide a measure of functional capability in ambulatory patients that is complementary to the 6MWT. The tests are reproducible, simple to administer, and have a documented response to therapeutic intervention with steroids.^17^,^19^,^23^ A stopwatch was used to time the 10-meter walk, standing from supine, and 4-stair climb and descent. Test–retest reliability of the TFTs in this study have been described elsewhere.^11^

#### Myometry

Upper and lower extremity strength assessment was performed using a myometer following standardized procedures.^24^,^25^ Muscle groups evaluated included knee flexors and extensors, elbow flexors and extensors, and shoulder abductors. Force in pounds was determined. These parameters were monitored during screening, at baseline, and every 6 weeks during treatment. Methodology and test–retest reliability of the strength measures have been described elsewhere.^11^

### Data Analysis

The longitudinal natural history changes over 48 weeks were analyzed from data obtained from the 57 placebo-control patients. For 6MWD, percent predicted 6MWD, TFTs, strength in pounds, mean and SD, change from baseline, and SD of change from baseline were determined. Data are reported as mean (SD) in the text and tables and displayed as mean ± standard error of the mean (SEM) in the figures. The statistical program used was SAS, version 9.3 (SAS Institute, Cary, North Carolina).

#### Percent Predicted 6MWD

To account for maturational effects such as age, height, and associated stride length,^5^,^6^,^10^ we calculated a percent predicted 6MWD according to the prediction equation described by Geiger and colleagues.^26^ This equation has been validated in DMD and is consistent with the findings of Henricson and colleagues^10^ using the same DMD-modified 6MWT protocol.

#### Time to Persistent 10% 6MWD Worsening

Time to persistent 10% 6MWD worsening was used to evaluate rates of disease stabilization and progression and was evaluated using Kaplan–Meier methods. The time to persistent 10% 6MWD worsening was defined as the last time that 6MWD was not 10% worse than baseline. For patients who did not have 10% 6MWD worsening or who were removed from the study, time to event was censored at the time of the last 6MWD value. Differences among strata were tested using a stratified log-rank test.

## RESULTS

### Patients' Characteristics

Patient characteristics of the group under evaluation (*N* = 57) are shown in Table 1. Of the 57 patients, 32 (56%) were <9 years of age, and 34 (60%) had baseline 6MWD of >350 m. All patients were males, ranging in age from 5 to 15 years (Table 1). All 3 premature stop codon types were represented in the study population. Forty of 57 DMD patients (70.2%) were on stable regimens of systemic glucocorticoids for ≥6 months at the initiation of the study, which consisted of either prednisone, prednisolone, or deflazacort used on either daily or intermittent schedules.

**Table 1:** Patient characteristics (evaluated at screening and baseline)

Demographics	Placebo treatment arm (*n* = 57)
Age, years	
Mean (SD)	8.3 (2.33)
Median	8.0
Range	5-15
Race, *n* (%)	
White	54 (94.7)
Black	0 (0.0)
Asian	1 (1.8)
Hispanic	1 (1.8)
Other	1 (1.8)
Body height, cm	
Mean (SD)	123 (11.8)
Median	122
Range	104-163
Body weight, kg	
Mean (SD)	29 (9.1)
Median	26
Range	16-55
Stop codon type, *n* (%)	
UGA	31 (54.4)
UAG	12 (21.1)
UAA	14 (24.6)

### 6MWT Compliance and Falls during the Test

As shown in Table 2, most patients were able to perform a valid 6MWT on their first and only attempt. The frequency of valid 6MWTs exceeded 98%. Fall data during the 6MWT from screening to the 48-week evaluation are shown in Table 2. In 93.1% of tests there were no fall events, and the median duration of 5 seconds per fall represents only 1.4% of the 6-minute evaluation time.

**Table 2:** Placebo DMD patients with missing data and falls during 6MWT by visit in Study 007 (*n* = 57)

Visit	Number of patients with missing 6MWT*	Tests (*n*)	Total falls (*n*) during 6MWT	Percent of tests with ≥1 falls	Median recovery time (seconds) per 6MWT taken for falls (range)
Screening	0	57	6	10.5	8.0 (2–44)
Baseline	0	57	5	8.8	3.0 (1–12)
Week 6	0	57	5	8.8	10.0 (7–26)
Week 12	1	56	2	3.6	4.0 (3–5)
Week 18	0	57	4	7.0	4.5 (3–6)
Week 24	0	57	2	3.5	3.5 (3–4)
Week 30	1	56	3	5.4	4.0 (3–23)
Week 36	4	53	2	3.8	14.0 (4–24)
Week 42	0	57	6	10.5	4.5 (2–17)
Week 48	2	55	0	—	—
	Total missed (percent of scheduled missed)	Total tests completed (percent completed)	Total falls	Total percent of tests with ≥1 falls	Median recovery time per 6MWT taken for falls
Totals	8 (1.6%)	505 (98.4%)	35	6.9%	5.0 (1-44)

6MWD, 6-minute walk distance.

* Missed tests = tests that were scheduled but not performed.

### Strength Changes by Myometry over 48 Weeks

**FIGURE 1:**
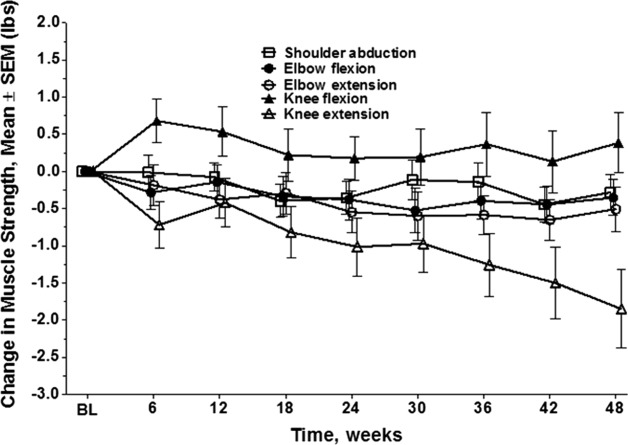
Change in strength for 5 strength measures by myometry obtained every 6 weeks for 48 weeks (mean ± SEM) in DMD patients (*n* = 57).

Mean strength changes over 48 weeks are shown for myometry in Figure 1. The mean baseline and mean change from baseline at 48 weeks were as follows: knee flexion, 11.06 and +0.38 pounds; knee extension, 12.96 and −1.85 pounds; elbow flexion, 8.14 and −0.35 pounds; elbow extension, 6.77 and −0.51 pounds; and shoulder abduction, 5.76 and −0.28 pounds. There were no significant differences in strength change over 48 weeks for glucocorticoid-treated versus non–glucocorticoid-treated patients.

### Changes in TFTs over 48 Weeks

**FIGURE 2:**
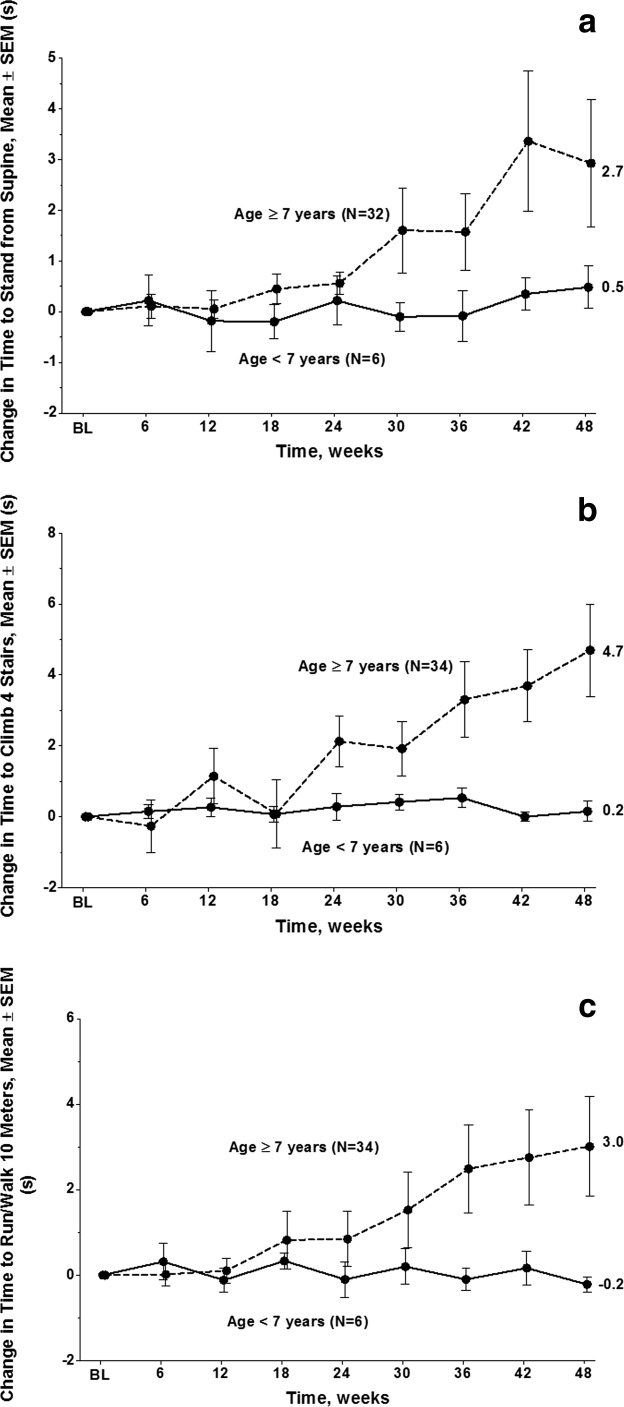
**(a)** Change in baseline time to stand from supine in seconds (mean ± SEM) for steroid-treated patients ≥7 years old (*n* = 32) and <7 years old (*n* = 6) with DMD over 48 weeks of evaluation. **(b)** Change in time to climb 4 stairs in seconds (mean ± SEM) for steroid-treated patients ≥7 years old (*n* = 34) and <7 years old (*n* = 6) with DMD over 48 weeks of evaluation. **(c)** Change in baseline time to run/walk 10 m in seconds (mean ± SEM) for steroid-treated patients ≥7 years old (*n* = 34) and <7 years old (*n* = 6) with DMD over 48 weeks of evaluation.

For the 40 patients on stable doses of glucocorticoids at the initiation of the study baseline the TFT results [mean (SD)] for 6 subjects <7 years and 34 subjects ≥7 years, respectively, were as follows: time to rise from supine = 4.0 s (1.13) and 12.9 s (12.33); time to climb 4 stairs = 2.5 s (0.67) and 6.6 s (6.30); time to run/walk 10 m = 4.8 s (0.86) and 7.1 s (2.80). Mean changes from baseline ± SEM over 48 weeks are plotted in Figure 2a–c. For all TFTs there was little change over 48 weeks in patients <7 years of age, whereas for patients ≥7 years there were greater changes in TFT values. In general, the SD and SEM of the change scores from baseline increase over time due to variability in disease progression.

### Relationship between Changes in 10-Meter Run/Walk and 6MWD

**FIGURE 3:**
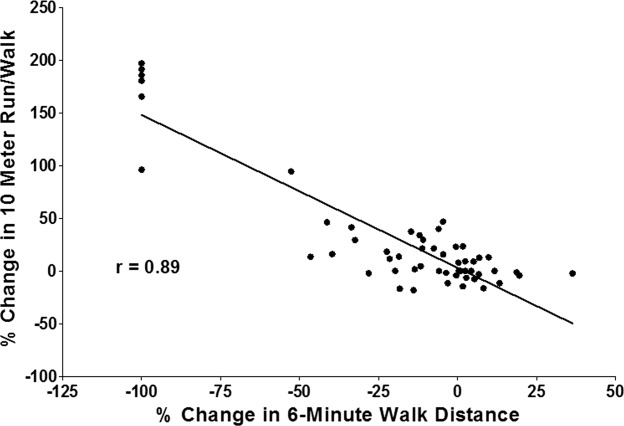
Relationship between percent change in 6MWD and percent change in 10-m run/walk test.

It is useful to evaluate the relationship between percent change in 10-m run/walk test and percent change in 6MWD to establish links between 6MWD data with longer-term 10-m run/walk data. Figure 3 shows a strong linear relationship between these 2 different types of ambulation measurement (*r* = 0.89, *P* < 0.00001).

### Relationship between Changes in 6MWD and Percent predicted 6MWD

There was a very high correlation between baseline 6MWD and baseline percent predicted 6MWD (*r* = 0.94). The correlation at 48 weeks between change from baseline in 6MWD and change from baseline in percent predicted 6MWD is *r* = 0.99. Thus, the percent predicted 6MWD data is not considered to be a separate endpoint but rather is both a maturational and normative context for the 6MWD, because the measure is strongly related to stride length and cadence and age in DMD patients and controls.^5^,^6^

### Natural History of 6MWD over 48 Weeks

**FIGURE 4:**
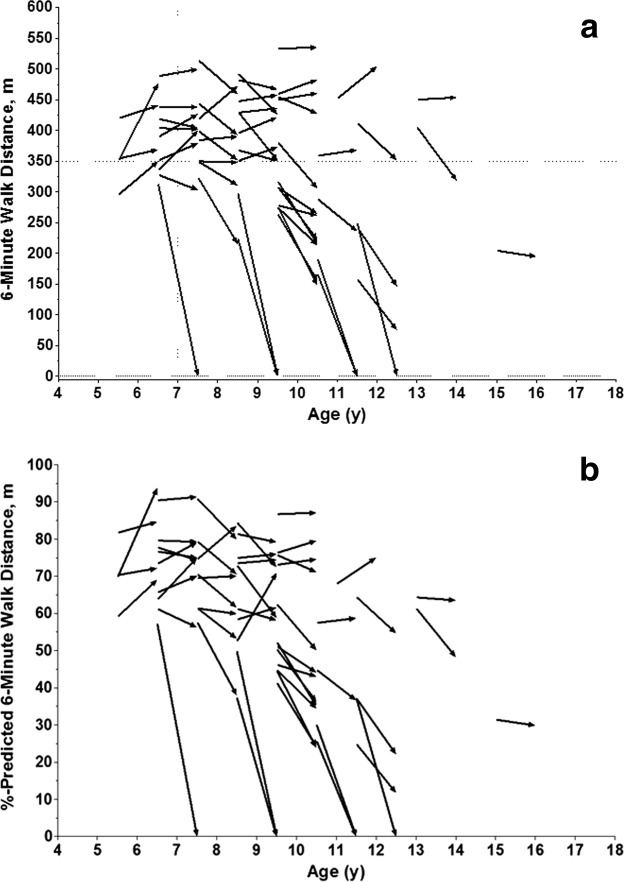
**(a)** Change in 6MWD over 48 weeks in 57 placebo-treated DMD patients. **(b)** Change in percent predicted 6MWD using age and height − calculated value over 48 weeks in 57 placebo-treated patients.

**FIGURE 5:**
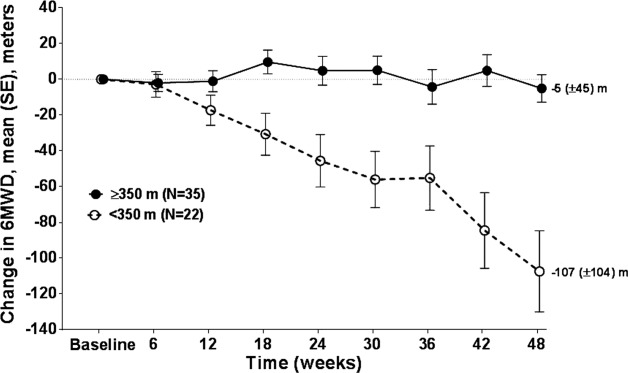
Change from baseline 6MWD in meters (mean ± SEM) over 48 weeks by baseline 6MWD level (<350 m vs. ≥350 m).

The 1-year natural history of changes in 6MWD and age- and height-based percent predicted 6MWD is shown in Figure 4a and b. Although differences between absolute values and percent predicted are obviously subtle it should be noted that a 6MWD of 450 m is approximately 80% predicted for a 6-year-old and 65% predicted for a 13-year-old. As shown previously,^5^,^6^ age and baseline walking ability are 2 important variables that determine decline in ambulation as measured by 6MWT; there is a tendency for the 6MWD to improve or be stable over the first 7 years of age,^5^,^6^,^8^,^9^ and patients who have lower initial 6MWD values and percent predicted 6MWD tend to show greater declines over the course of 48 weeks. Patients ≥7 years of age may show stable function or even improving function over 48 weeks, but they are almost always those with higher levels of baseline function (6MWD >350 m or percent predicted 6MWD >60%). Conversely, patients with baseline 6MWD <350 m demonstrate a greater decline in 6MWD over the 48-week evaluation period (Fig. 5).

Baseline 6MWD has prognostic significance, both for the primary assessment of bipedal ambulation and for evaluation of secondary assessments of activity, function, muscle strength, and fall frequency. The 57-patient cohort had a mean baseline 6MWD of 360 (SD = 88) m. The baseline range of 6MWD was 159–533 m, reflective of the heterogeneous nature of the patient population (*N* = 57, aged 5–15 years at study entry).

**FIGURE 6:**
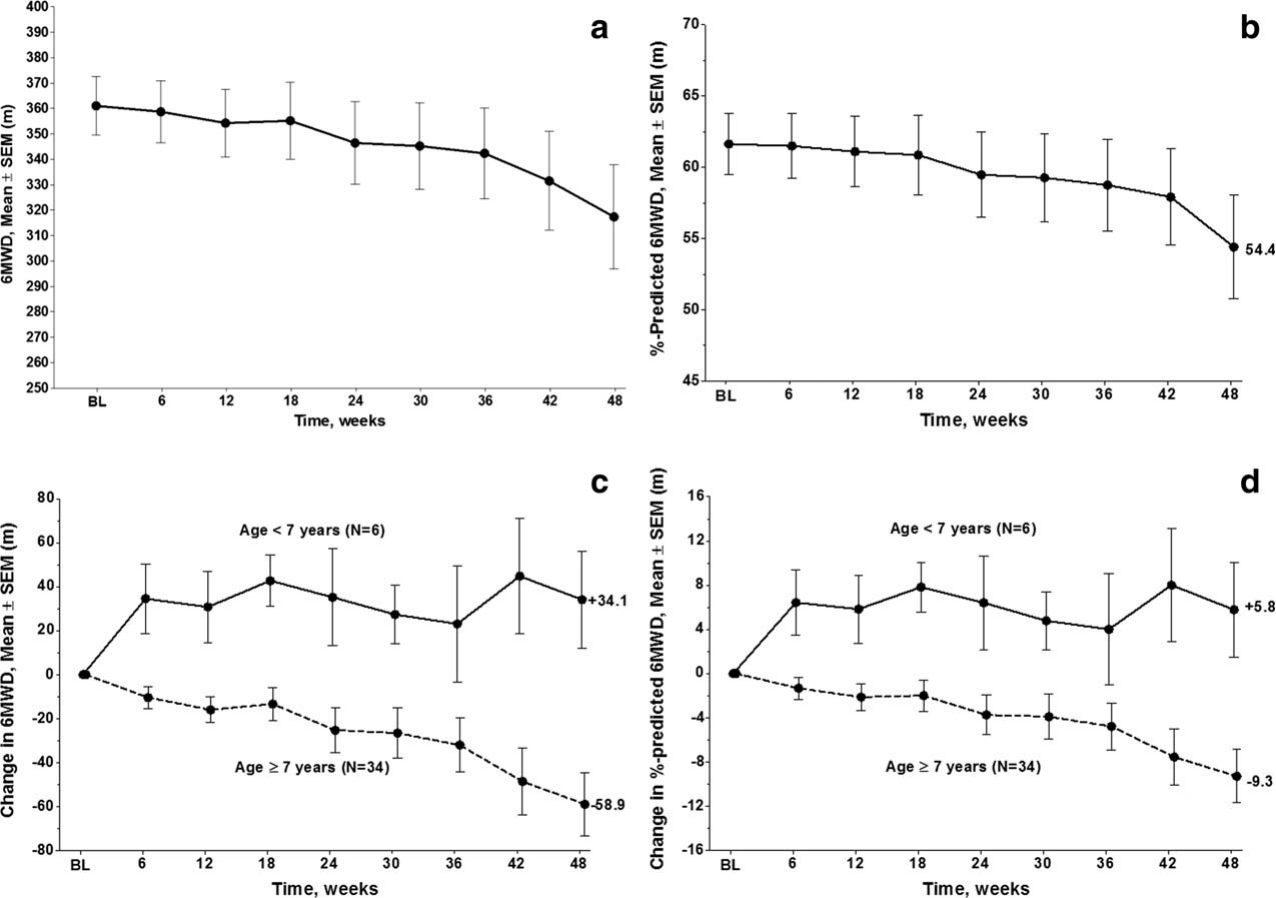
**(a)** 6MWD in meters (mean ± SEM) for all placebo-treated patients. **(b)** Percent predicted 6MWD (mean ± SEM) using age and height − calculated value for all placebo-treated patients. **(c)** Change in 6MWD in meters (mean ± SEM) for steroid-treated DMD patients <7 years old (*n* = 6) and ≥7 years old (*N* = 34). **(d)** Change in percent predicted 6MWD (mean + SEM) using age and height − calculated value for steroid-treated DMD patients <7 years old (*n* = 6) and ≥7 years old (*N* = 34).

**FIGURE 7:**
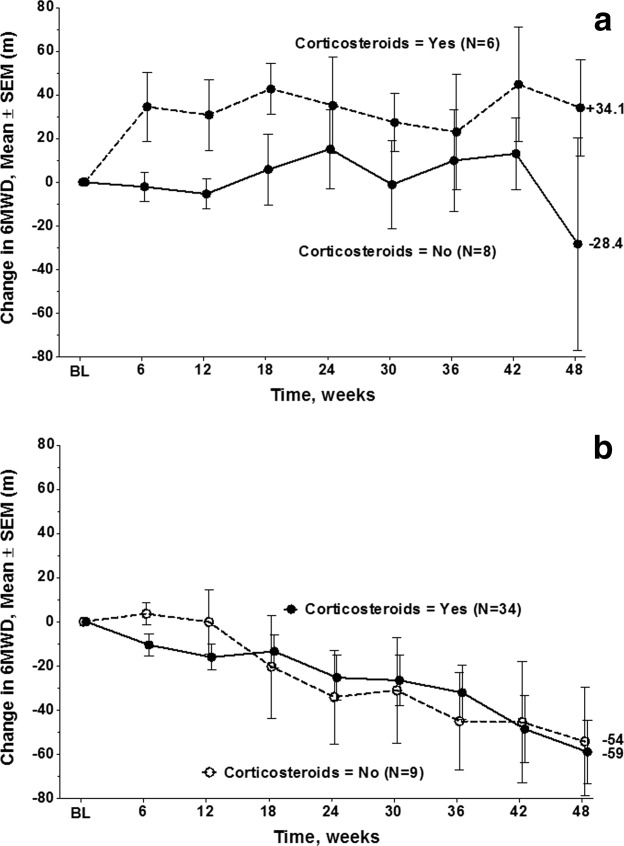
**(a)** 6MWD in meters (mean ± SEM) for DMD patients <7 years old treated with steroids (*n* = 6) and steroid-naive patients (*N* = 8). **(b)** 6MWD in meters (mean ± SEM) for DMD patients ≥7 years old treated with steroids (*N* = 34) and steroid-naive subjects (*N* = 9).

To assist clinical investigators with clinical trial design, we have provided the mean and SD from baseline to week 48 at 6-week intervals and the corresponding mean and SD change from baseline for both 6MWD and percent predicted 6MWD for all subjects (Table 3), steroid-treated patients (Table 4), and steroid-naive patients <7 years of age (Table 5). As seen in Figure 6a and b, there appears to have been be a progressive decline over 6-week intervals in mean 6MWD and predicted 6MWD. The effect of age on the progression of endpoints is shown for mean 6MWD (Fig. 6c) and mean percent predicted 6MWD (Fig. 6d) in steroid-treated DMD patients. The effect of long-term administration of glucocorticoid (≥6 months of stable dosing) on changes in ambulatory function (assessed as change in 6MWD at 6-week intervals over 48 weeks) is shown for <7-year-olds (Fig. 7a) and ≥7-year-olds (Fig. 7b). Although results are not statistically significant due to small numbers, the effect of long-term steroids on ambulatory function appears to have been greater for patients <7 years of age (Fig. 7a). In contrast, this long-term steroid effect was not observed in patients ≥7 years of age. Patients ≥7 years of age either on steroids (*n* = 34) or steroid-naive (*n* = 9) exhibited similar declines in 6MWD over 48 weeks (Fig. 7b).

**Table 3:** Natural history of 6MWD and percent predicted 6MWD in placebo-treated patients (steroid-treated and non-steroid-treated combined) evaluated every 6 weeks

≥5 years		Baseline	6 weeks	12 weeks	18 weeks	24 weeks	30 weeks	36 weeks	42 weeks	48 weeks
6MWD (m)	*n*	57	57	56	57	57	56	53	57	55
	Mean	361.1	358.8	354.3	355.2	346.5	345.3	342.4	331.5	317.4
	SD	87.5	92.4	100.0	113.9	122.9	127.6	130.4	146.6	152.3
	Mean Δ	0.0	–2.3	–7.2	–5.9	–14.6	–17.8	–22.5	–29.6	–44.1
	SD Δ	0.0	30.1	36.8	49.6	61.2	64.3	68.9	85.4	88.0
6MWD percent predicted	*n*	57	57	55	56	57	56	53	56	55
	Mean	61.6	61.5	61.1	60.9	59.5	59.3	58.8	57.9	54.4
	SD	16.3	17.3	18.3	21.0	22.5	22.9	23.4	25.3	27.0
	Mean Δ	0.0	–0.1	–0.9	–0.8	–2.2	–2.8	–3.5	–4.3	–7.3
	SD Δ	0.0	5.7	7.0	8.9	10.8	11.2	11.8	14.1	15.0

**Table 4:** The 48-week natural history data of 6MWD in placebo group patients (steroid-treated) evaluated every 6 weeks

		Baseline	6 weeks	12 weeks	18 weeks	24 weeks	30 weeks	36 weeks	42 weeks	48 weeks
<7 years 6MWD (m)	*n*	6	6	6	6	6	6	6	6	6
	Mean	382.8	417.3	413.5	425.5	418.0	410.1	405.9	427.6	416.9
	SD	42.7	55.1	30.7	30.7	32.9	25.9	35.6	40.6	35.1
	Mean Δ	0.0	34.5	30.7	42.7	35.2	27.4	23.1	44.8	34.1
	SD Δ	0.0	38.5	39.4	28.7	53.8	32.7	64.5	64.2	53.9
6MWD percent predicted	n	6.0	6.0	6.0	6.0	6.0	6.0	6.0	6.0	6.0
	Mean	72.1	78.6	78.0	80.0	78.5	76.9	76.1	80.2	77.9
	SD	6.8	9.4	6.7	5.9	7.7	5.1	8.8	9.8	8.3
	Mean Δ	0.0	6.4	5.8	7.8	6.4	4.8	4.0	8.0	5.8
	SD Δ	0.0	7.2	7.6	5.5	10.4	6.4	12.4	12.4	10.5
≥7 years										
6MWD (m)	*n*	34	34	34	34	34	33	31	34	33
	Mean	356.1	345.7	340.1	342.7	330.8	332.8	331.9	307.5	297.1
	SD	93.6	96.6	101.1	114.0	128.3	134.7	137.2	156.5	154.5
	Mean Δ	0.0	–10.5	–16.1	–13.4	–25.3	–26.5	–32.1	–48.6	–58.9
	SD Δ	0.0	29.2	33.8	42.8	59.0	66.1	68.5	88.1	81.9
6MWD percent predicted	*n*	34.0	34.0	33.0	33.0	34.0	33.0	31.0	34.0	33.0
	Mean	58.5	57.2	57.0	56.6	54.8	55.3	55.1	51.0	49.4
	SD	16.7	17.0	17.7	20.3	22.3	23.3	23.7	26.8	26.3
	Mean Δ	0.0	–1.3	–2.1	–2.0	–3.7	–3.9	–4.8	–7.6	–9.3
	SD Δ	0.0	5.8	7.0	8.2	10.5	11.7	11.8	14.9	13.9

**Table 5:** The 48-week natural history data of 6MWD in placebo group patients (non-steroid-treated) evaluated every 6 weeks

<7 years		Baseline	6 weeks	12 weeks	18 weeks	24 weeks	30 weeks	36 weeks	42 weeks	48 weeks
6MWD (m)	*n*	8	8	7	8	8	8	7	8	7
	Mean	366.3	364.2	364.4	372.1	381.5	365.3	368.6	379.4	341.4
	SD	63.5	62.1	63.4	73.8	80.0	91.2	90.7	81.1	163.9
	Mean Δ	0.0	–2.1	–5.4	5.8	15.1	–1.1	9.9	13.1	–28.4
	SD Δ	0.0	19.2	18.5	45.8	51.5	56.8	61.5	46.7	128.6
6MWD percent predicted	*n*	8	8	7	8	8	8	7	8	7
	Mean	70.1	69.3	69.2	70.8	72.6	69.4	69.9	71.9	64.7
	SD	11.5	11.2	11.6	14.1	15.7	17.5	17.2	15.2	30.7
	Mean Δ	0.0	–0.7	–1.3	0.8	2.5	–0.7	1.5	1.9	–5.8
	SD Δ	0.0	3.6	3.6	8.4	9.7	10.4	11.3	8.5	23.3

### Time to Persistent 10% 6MWD Worsening

**FIGURE 8:**
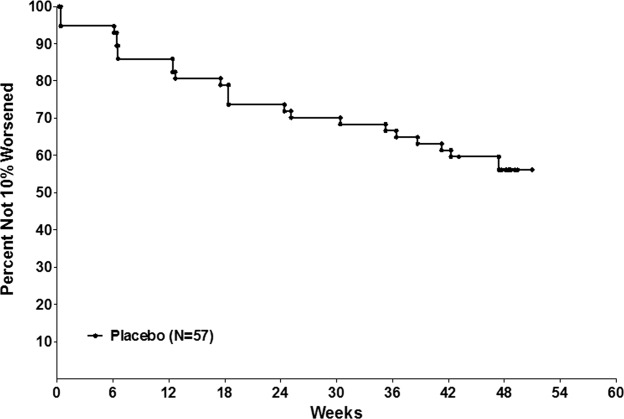
Time to persistent 10% 6MWD worsening in 57 DMD patients. Note that the relative proportion of patients who reached the 10% progression milestone was constant over 48 weeks (26.3% patients declined 10% or more during the first 24 weeks; 23.8% of the remaining patients declined 10% or more from weeks 24–48).

Using a time-to-event approach, the time to persistent 10% 6MWD worsening is shown in Figure 8. The relative proportion of patients who reached the 10% progression milestone appears to have been constant over 48 weeks (26.3% of patients declined 10% or more during the first 24 weeks, and 23.8% of the remaining patients declined 10% or more from 24 to 48 weeks). This time-to-event approach allows for determination of the time to disease progression relative to the patients' baseline values regardless of whether ambulation is lost and patients have transitioned to a wheelchair.

**FIGURE 9:**
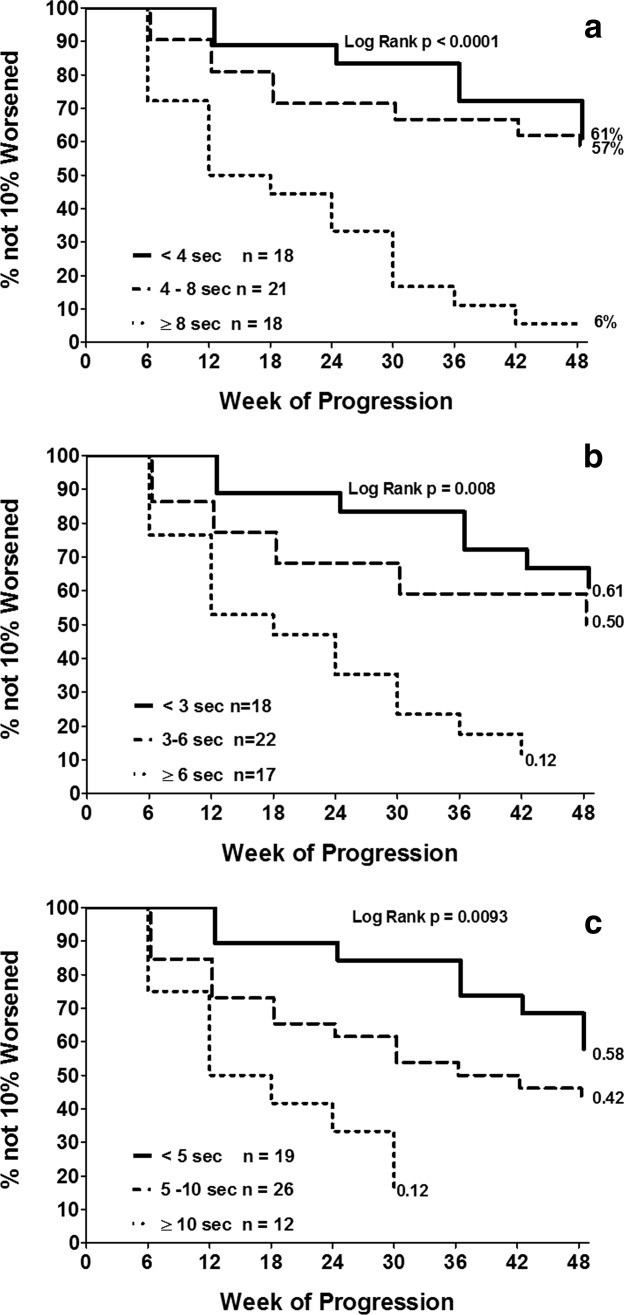
**(a)** Kaplan–Meier analysis of time to 10% progression in 6MWD by time to stand for 3 groups (time to stand <4 s, 4 to < 8 s, and ≥8 s/cannot stand). **(b)** Kaplan–Meier analysis of time to 10% worsening in 6MWD by time to climb 4 stairs for 3 groups (time to climb 4 stairs <3 s, 3 to <6 s, and ≥6 s/cannot climb). **(c)** Kaplan–Meier analysis of time to 10% worsening in 6MWD by time to run/walk 10 m for 3 groups (10-m run/walk <5 s, 5 to <10 s, and ≥10 seconds).

**FIGURE 10:**
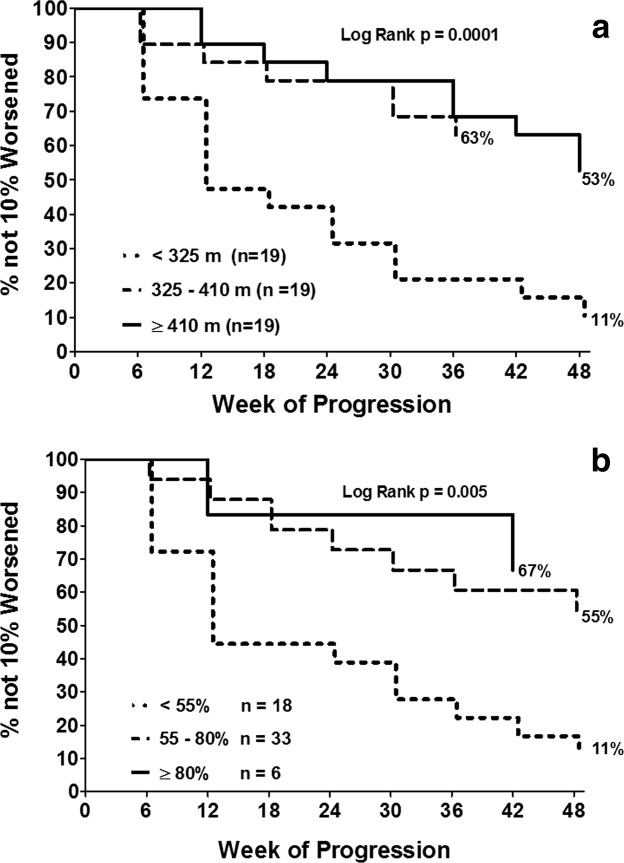
**(a)** Kaplan–Maier survival for time to 10% progression on 6MWD for 3 groups, based on initial 6MWD (<325 m, 325 to <410 m, and ≥410 m). **(b)** Kaplan–Meier analysis of time to 10% worsening in 6MWD based on initial percent predicted 6MWD at baseline for 3 groups (percent predicted 6MWD <55%, percent predicted 6MWD 55% to <80%, and percent predicted 6MWD ≥80%).

Figure 9a–c shows the effect of baseline TFTs on time to 10% progression in 6MWD. For all 3 TFTs there is a significant predicted value of time to perform the task at baseline and proportion progressing ≥10% on 6MWD. The effect of baseline 6MWD and percent predicted 6MWD on the proportion of patients who show persistent ≥10% decline in 6MWD over the subsequent 48 weeks is shown in Figure 10a and b. Initial 6MWD of <325 m and <55% predicted were associated with a greater likelihood of progressing ≥10% in 6MWD over 48 weeks.

### Prediction of Loss of Ambulation

Of the 57 patients, 15 (26.3%) did not have the ability to stand from supine (within the allowed 30 s) at the time of study entry. For the 15 who did not have the ability to stand from supine at baseline, 6 (40%) lost ambulation over 48 weeks. In contrast, only 1 of the 42 (2.3%) patients able to stand at baseline subsequently lost the ability to ambulate over the next 48 weeks (Fisher exact test, *P* < 0.0001). Six of 15 patients (40%) with baseline 6MWD <325 m lost ambulation over 48 weeks. In contrast, 0 of 42 patients with baseline 6MWD ≥325 m lost ambulation (Fisher exact test, *P* < 0.0001).

## DISCUSSION

Natural history data from this study regarding the 6MWT and other clinical endpoints over 48 weeks provide information that is critically important to the design of future clinical trials in ambulatory DMD. The 6MWT appears to be the most sensitive available endpoint for clinical trials of therapeutic agents targeted to stabilize function in ambulatory DMD subjects^6^,^11^ in the clinical setting, and TFTs also have a role as secondary endpoints in ambulatory DMD clinical trials. These data demonstrate that once ambulatory DMD patients deteriorate below a threshold 6MWD of 55% predicted or approximately 325 m, a high percentage have a decline of ≥10% in 6MWD over the subsequent year, or even lose ambulation. The threshold value of 6MWD for risk of more rapid deterioration (325 m) is ∽30 m below the mean 6MWD in our population of ambulatory DMD (358 m) patients and the mean 6MWD previously reported in DMD,^6^,^11^ which supports the clinical significance of a 30-m decline in 6MWD. The distribution-based methods used to calculate MCID that were applied to the 6MWD in DMD also support an MCID of ∽30 m.^11^ In dystrophin-deficient ambulatory boys there are important maturational and disease-severity issues and expected changes over 12 months of disease progression typically observed for specific endpoints. These are all critical factors to consider in trial design.

### Generalizability of Results

There have been no studies suggesting DMD patients with nonsense mutations in the dystrophin gene have a difference in disease course when compared with DMD patients with other types of mutations. The patients in this study have a 1-year decline in 6MWD similar to previously described DMD patients without nonsense mutations.^6^ Mazzone and colleagues reported a −42.3-m decline in 6MWD among DMD subjects >7 years of age. Those in this age range who were on either no steroids or intermittent regimens showed a −66.4-m decline, and those on daily steroids showed a −23.6-m decline.^9^ In another study, mean 12-month changes in 6MWD in a cohort in which 94% of subjects were treated with steroids was −44 (89) m among those with the TG/GG osteopontin SPP1 genotype and −8 (61) m among those with the TT SPP1 genotype.^27^ Although Becker muscular dystrophy (BMD) patients could theoretically have been included in our study, the study population was primarily DMD patients, given the inclusion criteria of more severe disease phenotype (i.e., onset of characteristic symptoms or signs of proximal weakness, waddling gait, and Gower maneuver by 9 years of age) and ongoing difficulty with ambulation at study entry.

There are currently no treatments for the underlying cause of DMD. Glucocorticoids (e.g., prednisone or deflazacort) are the only pharmacologic therapy demonstrated to be effective in temporarily reducing the decline in motor function in patients with DMD.^19^,^28^ Of note, in the natural history data from this study, the proportion of patients on glucocorticoids (70.2%) is similar to that of patients with DMD treated with glucocorticoids at baseline (210 of 340 = 62%) in the contemporary Cooperative International Neuromuscular Research Group (CINRG) natural history study.^29^ In the multistate population-based series from the U.S. Centers of Disease Control based on all patients (ambulatory and non-ambulatory) ∽50% were on glucocorticoids.^30^

### Implication of Maturational Issues and Severity of Disease at Baseline for Future Design of Clinical Trials

Studies have documented that the expected changes over time in both TFTs and 6MWD are strongly influenced by age, baseline ambulatory function, and maturational issues.^6^,^9^,^31^,^32^ The data we have presented on mean 6MWD, SD, mean change in 6MWD from baseline, and SD of the change in 6-week intervals over 48 weeks should be helpful for clinical trial planning. Stratification of patients is important to ensure the groups are balanced not only for age, but for baseline 6MWD and corticosteroid use.

The reliability of clinical measurement becomes more challenging in children <5 years of age. Our natural history data suggest that clinical trials in boys <7 years of age will need to: (a) be limited to therapeutic agents that will produce an effect of improving function rather than stabilizing function; (b) consider impairment values relative to age-matched controls; (c) be carried out over durations of >1 year so that disease progression may be measured; and/or (d) employ alternative candidate surrogate endpoints that can be measured reliably in young children.

In addition, we need to be mindful that some therapeutic agents in DMD are likely to require sufficient viable muscle fibers to be effective. For example, results published or presented from exon-skipping trials suggest that patients with better initial 6MWD (≥340–350 m or >50–55% predicted 6MWD estimated from data presented) may be more likely to demonstrate better response to treatment.^33^,^34^ Thus, the likelihood that a critical proportion of viable muscle fibers must be present for a treatment that induces more dystrophin expression to be efficacious needs to be balanced against the challenge of showing a treatment effect if the population is higher functioning at baseline and less likely to show deterioration over 12 months.

Useful serum surrogate biomarkers have not been identified for DMD, and both MR imaging biomarkers and measures of dystrophin expression on muscle biopsy have not been validated sufficiently to be employed as primary clinical trial endpoints. In the absence of an adequate surrogate endpoint, future clinical trials on the ambulatory DMD population need careful trial design with optimal inclusion and exclusion criteria (age, baseline 6MWD or percent predicted 6MWD, standing times, stable regimens of glucocorticoids, etc.), judicious choice of endpoints expected to be responsive to treatment, and adequate estimates of statistical power using clinically meaningful treatment effect sizes.

### Importance of Establishing the Connection between Change in 10-Meter Run/Walk and 6MWD

The 10-m run/walk is a measure that is easily obtained by the clinician in the outpatient clinic setting. We have shown that, on average, a 6-s performance on the 10-m run/walk (1.64 m/s) corresponds to 358 m on the 6MWT.^11^ In addition, a time of <6 s on the 10-m run/walk is associated with continued ambulation over the subsequent 12 months, and a time of >10–12 seconds is associated with a high risk of loss of ambulation over 12 months.^31^ Furthermore, data from the CINRG Duchenne Natural History Study demonstrated that a 10% decline in ambulatory function, as measured by the 10-m run/walk, is predictive of the number of months to loss of ambulation over 4 years.^29^,^32^ Thus, our current data, which show a high correlation between percent change in 10-m run/walk and percent change in 6MWD, suggest that a 10% change in ambulatory function, as measured by the 6MWT, would have similar clinical meaning in predicting loss of ambulation.

### Variability of Change in Endpoints over 48 Weeks

The results indicate that the 6MWT is, at present, the most reliable clinical endpoint requiring the smallest patient population for power. Previously there has been concern regarding the increasing standard deviation of the mean 6MWD, mean change in 6MWD in DMD over 48 weeks (Fig. 6), and the impact this increasing variability has on statistical power. It is important to note that all the TFTs also show increasing variability from baseline to 48 weeks, as measured by the standard deviation, standard error, and coefficient of variation, for mean values and mean change from baseline (Fig. 2). Strength measures using myometry also show increased SD and SEM values (Fig. 1) and mean change values over 48 weeks (not shown). Thus, the increasing variability is not an inherent problem with the clinical endpoints but rather represents the variability of disease progression in DMD that is an inherent aspect of the disease. This issue can be addressed by studying an appropriate sample size in clinical trials, calculated based on the variability of the endpoint. Further, it can be mitigated partly by including only patients on standard long-term steroid regimens and choosing inclusion criteria that produce more homogeneous populations.

### Strength Measures as Clinical Endpoints in DMD

The decline in strength was minimal over the course of the study. The greatest change in strength by myometry at 48 weeks, which occurred for knee extension, was lower than the previously calculated MCID for knee extension in DMD of 2.1 pounds (standard error of measurement method) and 2.4 pounds using the one-third of baseline SD method.^11^,^35^–^37^ Thus, in DMD a treatment that produces a therapeutic effect in strength over 48 weeks that would be greater than the MCID would at least need to produce small increases in strength in the functional muscle groups rather than reduction in decline in strength. Therefore, these data do not support the use of absolute values of strength by myometry as endpoints in ambulatory DMD unless the treatment under investigation is anticipated to produce a short-term *increase* in absolute strength values, such as that reported for glucocorticoids. An agent that stabilizes or slows decline in strength and would not be expected to increase strength, as anticipated for dystrophin restoration therapy, would require a longer duration trial. Part of the limitation in strength measurement is the lack of normative and percent predicted values for age. The other important limitation of strength measurement pertains to the floor effect of lower-extremity strength values typically seen in DMD patients ≥7 years of age, which are low at baseline^11^ and decline minimally over 48 weeks.

### TFTs as Clinical Endpoints in DMD

TFTs appear to have some potential utility in DMD clinical trials. TFTs that measure maximal burst activity are readily obtainable by the clinician in an outpatient setting, and they appear to predict the likelihood of a 10% progression in ambulatory function and loss of ambulatory function over the subsequent year (Fig. 9). It is not surprising that the threshold timed function values for predicting 10% progression in 6MWD are lower than the threshold values for predicting later loss of ambulation.^32^

Natural history data from CINRG and this study have shown that a standing time of >8 s predicts 10% progression over a year, but inability to stand (within 30 s) predicts a higher likelihood of loss of ambulation over 12 months. A >6-s time to climb 4 stairs is predictive of a greater likelihood of 10% progression in the 6MWD, whereas a >8-s stair climb predicts greater likelihood of loss of ambulation over 12 months.^32^ A ≥5-s 10-m run/walk time predicts time to 10% progression in 6MWD, whereas a >10–12-s 10-m run/walk time predicts loss of ambulation over 12 months.^31^,^32^

Ability to stand is a useful prognostic indicator for continued ambulation over 12 months, giving it utility as a potential inclusion criterion for a clinical trial. However, in trials of 12 months or shorter duration we would not recommend standing time as an endpoint for patients younger than age 7 years unless the therapeutic agent would be expected to improve standing time. The use of standing time as an endpoint in patients aged ≥7 years is limited by the significant proportion of subjects who cannot perform the functional task.

Whereas time to climb 4 stairs did not change for steroid-treated children <7 years of age, this measure showed a mean change of 4.7 ± 7.5 s for steroid-treated patients ≥7 years of age (greater than the MCID of 2.1–2.2 s for time to climb 4 stairs).^12^ Thus, stabilization of this measure over 12 months would be a clinically meaningful outcome. In addition, time to climb 4 stairs shows high test–retest reliability and the highest correlation (0.67) of any TFT measure with knee extension strength.^11^

Time to run/walk 10 m is a burst measure of ambulatory function rather than an endurance measure. Although the change over 12 months in 10-m run/walk was greater than the MCID of 1.4–2.3 s,^11^ the MCID/baseline mean ratio was much higher for the 10 m run/walk test (0.19–0.31) versus that for the 6MWD (0.08–0.09), and the test–retest reliability for the 10-m run/walk test was demonstrated previously to be lower than that for the 6MWT.^11^ Thus, as a clinical endpoint for trials, the 6MWD appears to have advantages as a more reliable and sensitive measure of change in ambulatory function compared with the shorter-interval 10-m run/walk test.

### Ambulation Declines more Precipitously in DMD Once a Threshold Level of Impairment Is Reached

In DMD, it is noteworthy that reduced 6MWD and percent predicted 6MWD values below 50–55% predicted (based on age and height) are associated with significant continued declines in ambulatory function as measured by the 6MWD, despite relatively small concomitant changes in knee extension strength values, which have reached a floor effect.^11^ In addition, with slowing of ambulation velocity and continued decrease in percent predicted 6MWD to <50–55% there is a precipitous increase in the energy cost of locomotion as measured by the Energy Expenditure Index.^11^ This may account for the more precipitous loss of ambulatory function that occurs in a significant percent of patients who have absolute 6MWD and percent predicted 6MWD values of <325 m and 50–55% predicted, respectively.

### A Change in 6MWD of 30 Meters Is Clinically Meaningful

Our data support the concept of a 30-m change in 6MWD being clinically meaningful. The MCIDs calculated from statistical distribution methods indicates that 30 m is a meaningful change.^11^ A ≥10% decline in ambulation over 12 months is associated with significantly greater likelihood of lost ambulation over the next 4 years^32^ and, given a typical baseline 6MWD of 350 m, a 30-m change to 320 m (which approaches a 10% change) places patients below a threshold level of function where they become at risk for losing ambulation. In addition, boys with DMD encounter considerable ambulatory challenges in their daily life that are clinically important. The distance of 30 m may have real-world significance in terms of keeping up with peers, distances required to get from a parking lot into a school, travel to a bus stop, or make it to a restroom. Data from placebo-controlled studies of laronidase for mucopolysaccharidosis type I (MPS I), idursulfase for MPS II, bosentan for primary pulmonary hypertension, and alglucosidase-α for Pompe disease also support the clinical meaningfulness of a 30-m treatment effect for the 6MWT.^37^–^40^ In these studies, differences in mean changes in 6MWD in drug-treated patients versus placebo-treated patients ranged from 28 to 44 m.^37^–^40^ The data from the trials in MPS I, MPS II, and Pompe disease are especially relevant given that the limitations on patient activity in these diseases and those in DMD result from disease-related impairments in both the neuromuscular and pulmonary systems. Strikingly, when accepted MCID thresholds of other diseases (as low as 5% change)^41^ are applied to the mean baseline 6MWD of this study, an 18-m change would be considered clinically meaningful. This further emphasizes that 30 m would be a robust measure of clinical meaningfulness in evaluation of 6MWD and would exceed the MCID for 6MWD from multiple accepted methodologies in other diseases.

In conclusion, in this study we have confirmed the 6MWT to be safe and feasible in DMD. Our findings support the clinically meaningful change in 6MWD to be in the range of 20–30 m, which can serve as the targeted treatment effect in 12-month trials in ambulatory DMD. Baseline 6MWD was found to be predictive of time to ≥10% worsening in function, a clinically meaningful milestone. A 10% decline in ambulatory function, as measured by the 10-m run/walk over 1 year, has been shown recently to be predictive of time to loss of ambulation over the subsequent 4 years.^35^ The close association between the percent change in time to run/walk 10 m and percent change in 6MWD also supports the notion that a 10% change in 6MWD is clinically meaningful and predictive of loss of ambulation. Finally, it appears that a decline of approximately 30 m from an average performance on the 6MWT in DMD to a threshold 6MWD of 325 m or 55% predicted would place a patient at risk for a more precipitous decline in ambulatory function over the subsequent year. Given the limitations of other measures in DMD, including surrogate biomarkers, strength by myometry, TFTs, and patient-reported outcome measures, the 6MWD is the recommended primary outcome measure in ambulatory DMD.

## APPENDIX 1

### PTC124-GD-007-DMD Study Group Collaborating Authors

**Australia**

*Murdoch Chidlren's Research Institute, Australia Royal Children's Hospital and University of Melbourne, Parkville, Victoria –* Monique Ryan, MBBS

*Department of Clinical Genetics, Children's Hospital at Westmead, New South Wales, Australia –* Kristi Jones, MBBS

**Belgium**

*Department of Pediatrics and Child Neurology, University Hospital Leuven, Belgium* – Nathalie Goemans, MD

**Canada**

*Department of Pediatrics, Children's Hospital Ontario, University of Western Ontario, London, ON, Canada –* Craig Campbell, MD

*Department of Neurology, Alberta Children's Hospital, University of Calgary, Alberta, Canada –* Jean Mah, MD

*Department of Pediatrics, Children's & Womens Health Center of British Columbial, University of British Columbia, Vancouver, BC, Canada–* Kathryn Selby, B.Sc. MBChB, MRCP, FRCPC

**France**

*Institiut de Myologie, Groupe Hospitalier Pitie-Salpetriere, Paris, France –* Pr. Thomas Voit

*Neurologie Pediatrique, Unite de Medecine Infantile Hopital d'Enfants, Marseilles, France –* Pr. Brigitte Chabrol

*Laboratoire d'Exploracion Fonctionnelles, Centre de Reference Maladies Neuromusculaires Nantez-Angers, CHU de Nantes, France –* Pr. Yann Pereon

**Germany**

*Department of Neuropediatrics, Neuromuscular Centre, University of Essen, Essen, Germany –* Dr. med Ulrike Schara

*Department of Pediatrics and Adolescent Medicine, University Hospital Frieberg, Germany –* Dr. Janberndt Kirschner

**Italy**

*Medicina Moleculare di Malattie Neuromuscolari e Neurodegenerative Dipartimento dei Laboratori Oespedale Pediatrico Bambino Gesu di Roma, Italy –* Prof. Enrico Bertini

*Dipartimento di Sciencze Pediatriche Medico-Chiurgiche e Neuroscienze dello Svilippo U.O. Complessa di Neurospichiatria Infantile Policlinica Universitario A. Gemelli, Rome, Italy –* Prof. Eugenio Mercuri

*Unita Operativa di Neurologia Fondazinone IRCCS, Ospedale Maggiore Policlinico, Milan, Italy –* Prof. Giacomo Comi

**Israel**

*Pediatric Neurology Unit, Hadassah Hebrew University Hospital, Jerusalem, Israel –* Prof. Yoram Nevo

**Spain**

*Departamento de Neurologia, Hospital Universitario La Fe, Valencia, Spain Hospital –* Juan Vilchez, PhD,

*Departamento de Neuropediatria, Hospital Sant Joan de Deu, Barcelona, Spain –* Jaume Colomer, PhD

**Sweden**

*Department of Pediatrics, Queen Sylvia Children's Hospital, University of Gothenburg, Sweden* – Mar Tulinius, MD, PhD,

*Department of Women's and Children's Health, Karolinksa University Hospital, Stockholm, Sweden –* Thomas Sejersen, MD, PhD

**United Kingdom**

*Institute of Genetic Medicine, Newcastle University, Newcastle Upon Tyne Great Britain –* Prof. Katherine Bushby

*Department of Neuroscience and Mental Health, King's College, London, UK King's College, London –* Prof. Francesco Muntoni

*National Hospital for Neurology and Neurosurgery, University College London Hospitals, London –* Dr. Rosaline Christina Mary Quinlivan

**United States**

*Department of Pediatrics and Neurology, Cincinnati Children's Medical Center, OH, USA –* Brenda Wong, MD, MBBS

*Department of Pediatrics, The Children's Hospital of Philadelphia, PA, USA –* Richard S. Finkel, MD

*Departments of Neurology and Pediatrics, University of Utah Medical Center, Salt Lake City, UT, USA –* Jacinda B. Sampson, MD, PhD, Kevin M. Flanigan, MD, Russell Butterfield, MD

*Department of Neurology, University of Minnesota, Minneapolis, MN, USA –* John W. Day, MD, PhD

*Department of Neurology, University of Iowa Children's Hospital, Iowa City, IA, USA –* Katherine Mathews, MD

*Department of Neurology, Children's Hospital Boston, MA, USA* – Basil T. Darras, MD, *Department of Rehabilitation Medicine, The Children's Hospital, University of Colorado, Denver, CO, USA –* Susan D. Apkon, MD

*Department of Neurology, The Children's Hospital, University of Colorado, Denver, CO, USA* –Julie Parsons, MD

*Department of Neurology, University of Kansas Medical Center, Kansas City, KS, USA* – Richard Barohn, MD

*Department of Neurology, Washington University School of Medicine at St. Louis, MO, USA –* Anne Connolly, MD,

*Department of Neurology, Children's Medical Center Dallas, University of Texas Southwestern, Dallas, TX, USA –* Susan Iannaccone, MD

*Department of Neurology, Columbia University Pediatric Neuromuscular Center, Columbia University Medical Center, New York, NY, USA–* Douglas M. Sproule, MD, Petra Kaufman, MD, MSc

*Department of Physical Medicine & Rehabilitation, Neuromuscular Medicine & Rehabilitation Research Center, University of California, Davis –*Jay Han, MD, Nanette Joyce, DO

*Northwest Florida Clinical Research Group, Gulf Breeze, FL, USA* – J. Ben Renfroe, MD

*Department of Neurology, Shriners Hospital for Children Portland, OR, USA –* Barry S. Russman, MD

*Department of Pediatrics and Division of Cardiology, Duke University, Durham, NC, USA* – Stephanie Burns-Wechsler, MD

*Department of Pathology, University of Iowa, Iowa City, IA, USA –* Steven A Moore, MD, PhD

*Department of Physiology, Unversity of Pennsylvania, PA, USA –* H Lee Sweeney, PhD

*OrthoCare Innovations, Mountlake Terrace, WA*, USA – Kim Coleman, MS

## Abbreviations

6MWD: 6-minute walk distance
6MWT: 6-minute walk test
BMD: Becker muscular dystrophy
DMD: Duchenne muscular dystrophy
MCID: minimal clinically important difference
TFT: timed function test
