# Supplementary material for: THE 6-MINUTE WALK TEST AND OTHER ENDPOINTS IN DUCHENNE MUSCULAR DYSTROPHY: LONGITUDINAL NATURAL HISTORY OBSERVATIONS OVER 48 WEEKS FROM A MULTICENTER STUDY
Source: Muscle Nerve. 2013 Jun 26;48(3):343–56. doi: 10.1002/mus.23902 (PMC3824082; doi:10.1002/mus.23902)
Supplement: Supplementary file 1 [file mus0048-0343-SD1.doc]

**Appendix 1: PTC124-GD-007-DMD Study Group Authors and Sub-Investigators**

**Collaborating Authors (Members of the PTC124 GD 007 DMD Study Group**

**Australia**

*Murdoch Chidlren’s Research Institute, Australia Royal Children’s Hospital and University of Melbourne, Parkville, Victoria –* Monique Ryan, MBBS

*Department of Clinical Genetics, Children’s Hospital at Westmead, New South Wales, Australia –* Kristi Jones, MBBS

**Belgium**

*Department of Pediatrics and Child Neurology, University Hospital Leuven, Belgium* – Nathalie Goemans, MD

**Canada**

*Department of Pediatrics, Children’s Hospital Ontario, University of Western Ontario, London, ON, Canada –* Craig Campbell, MD

*Department of Neurology, Alberta Children’s Hospital, University of Calgary, Alberta, Canada –* Jean Mah, MD

*Department of Pediatrics, Children’s & Womens Health Center of British Columbial, University of British Columbia, Vancouver, BC, Canada–* Kathryn Selby, B.Sc. MBChB, MRCP, FRCPC

**France**

*Institiut de Myologie, Groupe Hospitalier Pitie-Salpetriere, Paris, France –* Pr. Thomas Voit

*Neurologie Pediatrique, Unite de Medecine Infantile Hopital d’Enfants, Marseilles, France –* Pr. Brigitte Chabrol

*Laboratoire d’Exploracion Fonctionnelles, Centre de Reference Maladies Neuromusculaires Nantez-Angers, CHU de Nantes, France –* Pr. Yann Pereon

**Germany**

*Department of Neuropediatrics, University Clinic for Children, University of Essen, Essen, Germany –*  Dr. med Ulrike Schara

*Department of Neuropediatrics and Muscle Disorders, University Hospital Frieberg, Germany –* Dr. Janberndt Kirschner

**Italy**

*Medicina Moleculare di Malattie Neuromuscolari e Neurodegenerative Dipartimento dei Laboratori Oespedale Pediatrico Bambino Gesu di Roma, Italy –* Prof. Enrico Bertini

*Dipartimento di Sciencze Pediatriche Medico-Chiurgiche e Neuroscienze dello Svilippo U.O. Complessa di Neurospichiatria Infantile Policlinica Universitario A. Gemelli, Rome, Italy –*  Prof. Eugenio Mercuri

*Unita Operativa di Neurologia Fondazinone IRCCS, Ospedale Maggiore Policlinico, Milan, Italy –* Prof. Giacomo Comi

**Israel**

*Pediatric Neurology Unit, Hadassah Hebrew University Medical Center, Jerusalem, Israel –* Prof. Yoram Nevo

**Spain**

*Departamento de Neurologia, Hospital Universitario La Fe, Valencia, Spain Hospital –* Juan Vilchez, PhD,

*Departamento de Neuropediatria, Hospital Sant Joan de Deu, Barcelona, Spain –* Jaume Colomer, PhD

**Sweden**

*Department of Pediatrics, Queen Sylvia Children’s Hospital, University of Gothenburg, Sweden* – Mar Tulinius, MD, PhD,

*Department of Neuropediatrics, Karolinksa University Hospital, Stockholm, Sweden –* Thomas Sejersen, MD, PhD

**United Kingdom**

*Institute of Genetic Medicine, Newcastle University, Newcastle Upon Tyne Great Britain –* Prof. Katherine Bushby

*Department of Neuroscience and Mental Health, King’s College, London, UK King’s College, London –* Prof. Francesco Muntoni

*Robert Jones & Agnes Hunt Orthopaedic Hospital NHS Trust, Children’s Unit, Gobowen Oswestry –* Dr. Rosaline Christina Mary Quinlivan

**United States**

*Department of Pediatrics and Neurology, Cincinnati Children’s Medical Center, OH, USA –* Brenda Wong, MD, MBBS

*Department of Pediatrics, The Children’s Hospital of Philadelphia, PA, USA –* Richard S. Finkel, MD

*Departments of Neurology and Pediatrics, University of Utah Medical Center, Salt Lake City, UT, USA –* Jacinda B. Sampson, MD, PhD, Kevin M. Flanigan, MD, Russell Butterfield, MD

*Department of Neurology, University of Minnesota, Minneapolis, MN, USA –* John W. Day, MD, PhD

*Department of Neurology, University of Iowa Children’s Hospital, Iowa City, IA, USA –* Katherine Mathews, MD

*Department of Neurology, Children’s Hospital Boston, MA, USA* – Basil T. Darras, MD, *Department of Rehabilitation Medicine, The Children’s Hospital, University of Colorado, Denver, CO, USA –* Susan D. Apkon, MD

*Department of Neurology, The Children’s Hospital, University of Colorado, Denver, CO, USA* –Julie Parsons, MD

*Department of Neurology, University of Kansas Medical Center, Kansas City, KS, USA* – Richard Barohn, MD

*Department of Neurology, Washington University School of Medicine at St. Louis, MO, USA –* Anne Connolly, MD,

*Department of Neurology, Children’s Medical Center Dallas, University of Texas Southwestern, Dallas, TX, USA –* Susan Iannaccone, MD

*Department of Neurology, Columbia University Pediatric Neuromuscular Center, Columbia University Medical Center, New York, NY, USA–* Douglas M. Sproule, MD, Petra Kaufman, MD, MSc

*Department of Physical Medicine & Rehabilitation, Neuromuscular Medicine & Rehabilitation Research Center, University of California, Davis –*Jay Han, MD, Nanette Joyce, DO

*Northwest Florida Clinical Research Group, Gulf Breeze, FL, USA* – J. Ben Renfroe, MD

*Department of Neurology, Shriners Hospital for Children Portland, OR, USA –* Barry S. Russman, MD

*Department of Pediatrics and Division of Cardiology, Duke University, Durham, NC, USA* – Stephanie Burns-Wechsler, MD

*Department of Pathology, University of Iowa, Iowa City, IA, USA –* Steven A Moore, MD, PhD

*Department of Physiology, Unversity of Pennsylvania, PA, USA –* H Lee Sweeney, PhD *OrthoCare Innovations, Mountlake Terrace, WA*, USA – Kim Coleman, MS

**PTC124-GD-007-DMD Study Group – Sites and Sub-Investigators**

| **Principal Investigator Name and Site Location** | **Sub-Investigator Name** |
| --- | --- |
| **Australia** | |
| Dr. Monique M. Ryan, MBBS, FRACP, MMed  The Royal Children's Hospital Flemington Road Parkville, 3052 Victoria | Prof. Andrew J Kornberg, MBBS, FRACP  Dr. Victoria Rodriguez-Casero, MD  Dr. Alison Wray, MB, ChB  Mrs. Katheryn Carroll  Ms. Rachael Kennedy  Ms. Daniella Villano |
| Dr. Kristi J. Jones, MBBS, PhD, FRACP, DCH, CGHGSA  The Children's Hospital at Westmead Cnr Hawkesbury Rd and Hainsworth St. Locked Bag 4001 Westmead NSW 2145 | Prof Kathryn N North, BSc(Med), MBBS, FRACP, MD, AAN, CGHGSA  Dr. Mark Dexter (Med), MBBS (Hons I), FRACS  Ms. Stephanie Wicks  Mrs. Kristy Rose |
| **Belgium** | |
| Nathalie Goemans, MD  University Hospitals Leuven (UZ Leuven) Herestraat 49 3000 Leuven | Prof. Gunnar Marceo Buyse, MD  Marleen van den Hauwe  Bart Vrijsen |
| **Canada** | |
| Craig Campbell, MD, MSc  Children's Hospital E4-PMDU 800 Commissioners Rd E London, ON N6A 4G5 | n/a |
| Dr. Jean K. Mah, MD, MSc, FRCPC  Alberta Children's Hospital 2888 Shaganappi Trail NW Calgary, Alberta T3B 6A8 | Caitlin J. Wright  Angela Chiu  Lori M. Walker  Harvey B. Sarnat, MD, FRCPC |
| Dr. Kathryn Selby, MD,ChB,MRCP,FRCPC  Children’s & Women’s Health Centre of British Columbia & University of British Columbia 4480 Oak Street Vancouver, BC, V6H 3V4 | Carol King. BScPT  Lorelyn Meisner. BScPT |
| **France** | |
| Pr. Thomas Voit  Groupe Hospitalier Pitie-Salpetriere 47/83 boulevard de I'Hopital 75013 Paris France, and Groupe Hospitalier Armand-Trousseau 26, avenue du Docteur Arnold-Netter 75012 Paris | Dr. Valérie Doppler  Dr. Denis De Castro  Mrs. Valérie Decostre PT |
| Pr. Brigitte Chabrol  Unité de Médecine Infantile Hôpital d'Enfants CHU Timone Bd Jean Moulin 13385 Marseille Cedex 5 | Pr. Nicolas Levy  Dr. Cécile Halbert |
| Pr. Yann Pereon  Laboratoire d'Exploration Fonctionnelles Hotel-Dieu Place Alexis Ricordeau 44093 Nantes Cedex 1 | Dr. Armelle Magot  Dr. Julie Perrier  Dr. Jean-Yves Mahe, PT  Mrs. Anne-Sophie Perrau, PT |
| **Germany** | |
| PD Dr. med Ulrike Schara  Dept of Neuropaediatrics University of Essen - University Clinic for Children Hufelandstr. 55, 45122 Essen | Dr. med. Sören Lutz  Dr. med. Melanie Busse  Dr. med. Adela Della Marina  Thomas Bosbach, PT |
| Dr. Janbernd Kirschner  University Hospital Dept of Neuropediatrics and Muscle Disorders Mathildenstr. 1 79106, Freiburg | Dr. med. Angela Stanescu  Dr. med. Annette Pohl  Dr. med. Cornelia Rensing-Zimmerman  Ulrike Eisele  Ina Fetzer  Sibylle Vogt |
| **Italy** | |
| Prof. Enrico Bertini  U.O Medicina Molecolare di Malattie Neuromuscolari e Neurodenerative Dipartimento dei Laboratori Ospedale Pediatrico Bambino Gesu Piazza S. Onofrio, 4 00165 Roma | Dr. Adele D’Amico  Dr. Annamaria Kofler  Dr. Adelina Carlesi  Dr. Anna Maria Bonetti  Maria Giulia Gagliardi, MD, PhD  Dr. Luigino Santecchia  Dr. Francesco Emma  Dr. Gianluca Bergami |
| Prof. Eugenio Maria Mercuri  Dipartimento di Scienze Pediatriche Medico-Chirurgiche e Neuroscienze dello Sviluppo U.O. Complessa di Neuropsichiatria Infantile Policlinico A. Gemelli-Università Cattolica Sacro Cuore Largo Gemelli 8 00168 Roma  Ospedale Pediatrico Bambin Gesu Piazza S. Onofrio 4, 00165 Roma | Dr. Gessica Vasco  Dr. Flaviana Bianco  Dr. Elena Stacy Mazzone  Dr. Roberto De Sanctis  Dr. Paolo Alfieri  Dr. Marika Pane  Dr. Sonia Messina |
| Prof. Giacomo Pietro Comi  Unita Operativa di Neurologia Fondazione IRCCS Ospedale Maggiore Policlinico, Mangiaglli e Regina Elena Via Francesco Sforza, 35, 20122 Milano | Dr. Francesca Magri  Dr. Valeria Lucchini  Dr. Stefania Paola Corti  Dr. Maurizio Gualtiero Moggio  Dr. Monica Sciacco  Prof. Nereo Bresolin  Dr. Alessandro Cesare Prelle  Dr. Roberta Magri  Dr. Roberta Virgilio  Dr. Costanza Lamperti |
| **Israel** | |
| Prof. Yoram Nevo  Hadassah Medical Center Mount Scopus P.O. Box 24035 Jerusalem | Dr. Talia Dor-Wollman  Adina Bar-Lev  Debbie Krojanker-Yaffe  Elana Weisband |
| **Spain** | |
| Juan J Vilchez, PhD  Hospital Universitario La Fe Neurology/Neuropediatric Department Avda. Campanar, 21 46009 Valencia | Nuria Muelas, MD  Teresa Sevilla, PhD  Patricia Smeyers, PhD  Florencio Calle  María González  Alberto de la Osa, MD  Christina Bejar |
| Jaume Colomer, PhD  Hospital Sant Joan de Deu Neuropediatric Department Passeig Sant Joan de Deu, 2 08950 Esplugues de Llobregat Barcelona | Carlos Ignacio Ortez, PhD  Andrés Nascimento, PhD  Ana Febrer, PhD  Julita Medina, MD  Robert Muni |
| **Sweden** | |
| Mar Tulinius, MD, PhD  Avdelningen for Pediatrik Sahlgrenska Akademin vid Goteborgs Universitet Drottning Silvias Barn-och Ungdomssjukhus 416 85 Göteborg | Brynja Thorarinsdottir, MD  Niklas Darin, MD  Ulrica Sterky, RN  Anna-Karin Kroksmark, PT  Lisa Berglund, PT |
| Thomas Sejersen, MD, PhD  Neuropediatriken Astrid Lindgrens Barnsjukhus Kaolinska Universitetssjukhuset, Solna 171 76 Stockholm | Mia Hovmöller, MD  Erika Trulsson, RN  Agneta Hök, PT  Pernilla Kipping, PT |
| **United Kingdom** | |
| Prof. Katherine M. D. Bushby  Clinical Research Centre 4th Floor Leazes Wing Royal Victoria Road NE1 4LP, Newcastle Upon Tyne | Professor Volker Straub  Dr. Guglieri Michela  Dr. Anna Sàrközy  Dr. Tracey Willis  Dr. Michelle Eagle  Dr. Anna Mayhew  Michelle McCallum |
| Prof. Francesco Muntoni  The Somers Clinical Research Facility (CRF) Great Ormond Street Hospital for Children NHS Trust Level 1, Frontage Building Great Ormond Street London, WC1N 3JH | Dr. Adnan Yousaf Manzur  Dr. Stephanie Ann Robb  Marion Main  Maria Ash  Sofia Lampropoulou  Dr. Maria Kinali |
| Dr. Rosaline Christina Mary Quinlivan  Robert Jones & Agnes Hunt Orthopaedic Hospital NHS Trust, Children’s Unit, Gobowen Oswestry, SY10 7AG | Dr. Martin Richard Smith  Dr. Rajesh Pandey  Suzanne James  Nick Emery  Lynne Groves  Richa Kulshrestha |
| **United States** | |
| Brenda L. Wong  Cincinnati Children's Hospital Medical Center 3333 Burnet Avenue MLC 2015 Cincinnati, OH 45229 | James Collins, MD  Michelle McGuire  Ann McCormick  Paula Morehart  Shengyong Hu  Rebeccah Brown |
| Richard S. Finkel, MD  The Children's Hospital of Philadelphia 34th and Civic Center Blvd. Philadelphia, PA 19104 | Carsten G Bonnemann, MD  Michele L Yang, MD  Aileen Reghan Foley, MD  Laureen Murphy-Kotzer, RPh  Lindsay Dorsey, BSN, RN  Timothy Estilow, OTR/L  Allan Glanzman, PT, DPT, PCS, ATP  Allison Paisley, BSN, RN  Sabrina Yum, MD  Teesha Thomas, BSN, MPH  Karna Smith, PT |
| Jacinda B. Sampson, MD, PhD  Kevin M. Flanigan, MD  University of Utah Medical Center 50 North Medical Drive Salt Lake City, UT 84132 | Eduard Gappmaier, PT, PhD  Mark B Bromberg, MD  Brianna McGerty, BS  Lauren Lahdan Heidarian, BS  Kathryn Swoboda, MD  Victor Gappmaier |
| John W. Day, MD, PhD  University of Minnesota Clinical and Translational Science Institute 717 Delaware Street SE, Room 205 Minneapolis, MN 55414 | Peter Isaak Karachunski, MD  Marcia Margolis, PT  Amy Erickson  Joline Dalton |
| Katherine Dianne Mathews, MD  Institute for Clinical and Translational Science Clinical Research Unit 3 South, General Hospital, UIHC 200 Hawkins Drive Iowa City, IA 52242 | Daniel Joseph Bonthius, MD  Carrie M Stephan, RN  Karla Sue Laubenthal, MD |
| Basil T. Darras, MD  Children's Hospital Boston 300 Longwood Avenue Boston, MA 02115 | Peter B. Kang, MD  Susan O Riley, PT, MS, DPT, PCS  Janet Quigley, PT, PCS  Hailly Butler, BS |
| Julie Parsons, MD  Susan D. Apkon, MD  The Children's Hospital 13123 East 16th Avenue Aurora, CO 80045 | Melissa Gibbons, MS  Terri Carry, PT |
| Richard J. Barohn, MD  Landon Center on Aging University of Kansas Medical Center 3599 Rainbow Boulevard Kansas City, KS 66160  University of Kansas Medical Center 3901 Rainbow Boulevard Kansas City, KS 66160 | Majed J. Dasouki, MD  Heather S. Anderson, MD  Anne Arthur, MS, ARNP, BC  Jeffrey Murray Burns, MD  Mazen M. Dimachkie, MD  Mamatha Pasnoor, MD  Yunxia (Insia) Wang, MD  Laura Herbelin, BS  Rosemary Myles |
| Anne Connolly, MD  Washington University 517 S. Euclid Avenue Box 8111 St. Louis, MO 63110 | Alan Pestronk, MD  Muhammad Al-Lozi, MD  Glenn Lopate, MD  Paul Golumbek, MD  R. Brian Sommerville, MD  Leo Wang, MD  Anna Wojcicka-Mitchell, MD  Andrew Godbey, MD  Betsy Malkus, PT  Jeanine Schierbecker, PT  Catherine Siener, PT  Mei Lu, MD, PhD  Matthew Harms, MD  Arun Varadachary, MD, PhD  Stanley Iyadurai, MD, PhD  Luisa Rojas, MD |
| Susan T. Iannacone, MD  Children’s Medical Center Dallas Ambulatory Care Pavilion 2350 Stemmons Freeway Dallas, TX 75207 | Heather Gilbreath, PA-C  Chaiyos Khonghatithum, MD  Ruth Merryman, PharmD  Leslie Nelson, MPT  Mariam Andersen, MA, CCRP  Medrith Greene, RN, BSN |
| Douglas M. Sproule, MD  Petra Kaufmann, MD, MSc  Columbia University Pediatric Neuromuscular Center 180 Fort Washington Ave. New York, NY 10032 | Darryl De Vivo, MD  Vanessa Battista, RN, MS, CPNP, CCRC  Andre Constantinescu, MD, PhD  Jacqueline Montes, PT, MA, NCS  Sally Dunaway, PT  Megan Montgomery, BS  Jonathan Marra, BS |
| Craig M. McDonald, MD  Department of Physical Medicine & Rehabilitation,  Neuromuscular Medicine & Rehabilitation Research Center  University of California, Davis Medical Center  4860 Y Street, Suite 3850, Sacramento, CA 95817 | Jay Han, MD  Erik Henricson, MPH  Richard Ted Abresch, MS  Nanette Joyce, DO  Erica Goude, BA  Linda Johnson, PT  Alina Nicorici, BS  Michelle Cregan |
| J. Ben Renfroe, MD  Northwest Florida Clinical Research Group 400 Gulf Breeze Parkway Suite 203 Gulf Breeze, FL 32561 | Genei Bougher, ARNP  Theresa Keohane, ARNP |
| Barry S. Russman, MD  Shriners Hospitals for Children 3101 SW Sam Jackson Park Road Portland, OR 97239-3009 | Michael D Sussman, MD  Kirsten Zilke, PT  Eduardo del Rosario, MSN, FNP-C |
| Stephanie Burns-Wechsler, MD  Duke Clinical Research Unit Rankin Ward, 2nd Floor, Duke Clinics Trent Drive Durham, NC 27710 | Vern C Juel, MD  Lisa Hobson-Webb, MD  Edward C. Smith, MD  Joanne Mackey, CPNP  Laura Case, DPT  Kathleen Ollendick, PT |
